# Symmetries and ground

**DOI:** 10.1007/s11098-024-02133-7

**Published:** 2024-04-29

**Authors:** Martin Glazier

**Affiliations:** Scripps College, Claremont, CA USA

**Keywords:** Ground, Symmetry, Qualitativism, Relationalism, Tense, Quantity, Fragmentalism

## Abstract

If the tiles of a mosaic are arranged symmetrically, then the image those tiles constitute must be symmetric as well. This paper formulates and defends the general principle at work in this case: roughly, that a symmetry cannot ground an asymmetry. It is argued that the principle supports strong objections to four metaphysical views: qualitativism, relationalism, the tenseless or ‘B’ theory of time, and comparativism. A response to these objections is developed which appeals to fragmentalism, the view that reality contains facts which are incompatible with one another. But fragmentalism might be thought too bizarre to tolerate, and if it is, then the objections developed in this paper may well be fatal.

## Introduction

Stuck at home during yet another lockdown, I determine to develop my artistic side. I create a mosaic—a depiction of a sunflower—by arranging thousands of equally-sized, round, colored tiles in a special way. I first lay down a brown tile to serve as the center of the flower. I then expand outward in concentric circles, placing tiles of the same color at regular intervals during each circumnavigation. As I reach the flower’s petals, the tiles become a brilliant yellow. Once every tile is in its place, I have my sunflower.

Just as the colored tiles repeat as one moves in a circle around the center, so does the image constituted by those tiles. If these tiles here depict one of the sunflower’s petals, for instance, then one will encounter perfectly similar petals again and again as one moves clockwise or counterclockwise.

The tiles have a kind of spatial symmetry to them: a rotational spatial symmetry. And in virtue of that symmetry, the image those tiles constitute must be rotationally symmetric as well.

But wait: do I really want to be a mosaicist? Perhaps I should take up origami instead. I fold and refold a sheet of paper until it takes the shape of a crane. Viewing the folded sheet from above, I can see that its left half perfectly mirrors its right half. But it is not only the two halves of the folded sheet which mirror one another; the two halves of the crane constituted by that sheet mirror one another as well. The folded sheet of paper has a kind of spatial symmetry: a reflectional spatial symmetry. And in virtue of that symmetry, the crane must be reflectionally symmetric as well.

Then again, maybe I should work on my frieze patterns. On a long, horizontal strip of paper, I draw a repeating sequence of line segments at right angles to one another, producing a winding, decorative border of the sort sometimes seen on the pottery of ancient Greece or the takeaway coffee cups of New York City. If I could extend my drawing infinitely in both directions, its line segments would exhibit a kind of spatial symmetry: a translational spatial symmetry. And in virtue of that symmetry, the decorative border constituted by those line segments would be translationally symmetric as well.

Or maybe I should leave the visual arts aside altogether and get back to practicing the piano. I decide to learn the *Moonlight Sonata*. It opens with a repeating melody: the same three pitches in the same order, again and again. Due to this repetition, the sounds produced by the piano exhibit a kind of *temporal* symmetry: a translational temporal symmetry. Or rather, they would do so if they repeated eternally in both temporal directions. Such sounds have the temporal equivalent of the spatial symmetry found in a frieze pattern. And it is in virtue of that temporal translational symmetry that the melody constituted by the sounds is translationally symmetric as well.^1^

These and similar examples suggest that some sort of highly general principle, applicable in both spatial and temporal cases, links the notion of symmetry to the notion of constitutive or in-virtue-of dependence. With many others, I understand this form of dependence in terms of the notion of *ground* (Rosen, 2010; Fine, 2012a). In this paper, I formulate and defend the symmetry principle at work in the above cases (Sect. 2) and investigate its consequences. I argue that the principle supports powerful objections to the following four reductionist views (Sect. 3):

1. The facts about particular individuals are grounded in the purely qualitative facts (*qualitativism*).
2. The facts about points and regions of space are grounded in the facts about the spatial relations between material bodies (*relationalism*).
3. The tensed facts are grounded in the tenseless facts (the *tenseless *or* ‘B’ theory of time*).
4. The facts about intrinsic quantities are grounded in the facts about quantitative relations (*comparativism*).

I believe the symmetry principle has applications beyond these four views, including applications to personal identity and to the metaphysics of subjectivity. But there is no room here for an exhaustive discussion of the principle’s applications and so I will limit myself to the four views listed above. In addition to their intrinsic interest, these applications show just how broad the ramifications of the principle are.

The objections I will develop are powerful, but they do admit of a response (Sect. 4). The response appeals to what Fine (2005) has called *fragmentalism*, the view that reality contains facts which are incompatible with one another. But this is a very strange view, one which Fine himself concedes ‘might even be regarded as metaphysically repugnant’ (307). And if the fragmentalist response is ultimately too bizarre to tolerate, then I believe the objections of Sect. 3 are decisive.

But we must be clear on those objections’ targets. The symmetry principle does not threaten every view that might reasonably be called qualitativist, relationalist, and so on. It threatens only non-eliminativist versions of those views. Defenders of eliminativist views have nothing to fear (from the symmetry principle at least).

To understand the difference between eliminativism and non-eliminativism, take relationalism as an example. The eliminativist relationalist denies that there are any facts at all about points and regions of space, perhaps holding that our apparent reference to such things is a mere artifact of our representations of the physical world and that these things do not really exist. The only facts there are, for her, are facts about material bodies and the spatial relations between them. The non-eliminativist, by contrast, is willing to concede that there are facts about points and regions. She will take it to be a genuine fact, for example, that the region I occupy is smaller than that occupied by the Eiffel Tower. But she will insist that such facts are not fundamental; rather, they are grounded in other facts, namely facts about the spatial relations between material bodies.^2^ It is this ground-based, non-eliminativist form of relationalism that is my target—and similarly for the other three views I will discuss.

## The symmetry principle

### The principle stated

Let us say that a grounding explanation is one in which we explain some fact by saying how it is grounded. I take the examples given at the outset of this paper to motivate something like the following principle:

*No Asymmetry from Symmetry (first pass)* In a grounding explanation, if the explanans is symmetric, the explanandum will also be symmetric.

But this rough statement of the principle needs considerable refinement.

Start with the notion of explanation involved in the principle. I adopt a conception on which the constituents of an explanation are worldly items like facts or states.

There is a familiar distinction between full and partial explanation. The principle must be taken to involve *full* explanation, since it is false if taken to involve partial explanation.

To see why, return to my rotationally symmetric sunflower mosaic, and suppose I implement the following garish change: I replace just one of the yellow tiles, in just one of the flower’s petals, with a bright orange tile. Then the tiles constituting the center of the flower will remain rotationally symmetric, and the arrangement of those tiles will *partly* explain the state of the flower-image as a whole, yet the latter will no longer be symmetric. Do we here have a counterexample to the principle? Not if the principle involves full explanation, for the orange tile renders the full explanans rotationally asymmetric.

A less familiar distinction is between pruned and unpruned explanation. The explanandum of an unpruned explanation contains everything that is explained by the facts to which the explanation appeals, whereas the explanandum of a pruned explanation contains only some of what is so explained. The symmetry principle must be understood to involve *unpruned* explanation, for it is false if understood to involve pruned explanation.

To see why, consider the mosaic again (but without the orange tile this time). Although the arrangement of the tiles (fully) explains the state of the left half of the flower-image, here the explanans is symmetric but the explanandum is not. Counterexample? Not if the principle involves unpruned explanation, since the unpruned explanandum concerns not only the image’s left half but its right half too.

We must also clarify the notion of symmetry involved in the principle. The principle is in fact more powerful than it may have appeared. For it allows us to infer not just from some form of symmetry or other to some form of symmetry or other, but from a particular form of symmetry to that same form. Recall the examples given at the outset of this paper. In the case of the sunflower mosaic, the relevant form of symmetry is rotational. The case of the origami crane, by contrast, involves reflectional symmetry. And the cases of the frieze pattern and of the *Moonlight Sonata* involve translational symmetry, with the former involving spatial translational symmetry and the latter temporal translational symmetry. The application of the principle to particular forms of symmetry will be important in what follows.

We can be even more specific than we have been so far about the form of symmetry something has. For example, the origami crane exhibits not just reflectional symmetry but a certain particular form of such symmetry: it is symmetric about the plane which contains the crane’s beak and tail and which is orthogonal to the line containing its wingtips. The principle entails that, because the folded paper possesses this particular form of reflectional symmetry, the crane must possess it as well. Unless it matters, however, we will usually not bother to fully specify the relevant particular form of symmetry.

But when should we say that something *has* a given form of symmetry? Quite often we can appeal to intuition. It is intuitively clear, for instance, that the symbol  has reflectional symmetry and that  has rotational symmetry.

But we need not rest only on intuition; we can say more. One way to say more would be to offer a definition of symmetry. I will not try to do that in this paper. Instead, I will rely on a standard way of thinking, familiar from mathematics and empirical science, on which symmetry is linked with invariance under transformation.^3^ Each form of symmetry is taken to correspond to a certain transformation: rotation, reflection, translation, and so on. Let us take a *state* to be any actual or possible fact (I will use ‘state’ and ‘fact’ interchangeably in what follows). We may then give the following initial statement of the link between symmetry and invariance:

*Symmetry–invariance link (first pass)* The state *a* has a given form of symmetry just in case it is invariant under the corresponding transformation *T*—i.e., just in case $a=Ta$.

(This statement of the link will be revised in Sect. 2.3.) I offer this link not as a definition of symmetry but simply as a true principle that can often help us to judge whether a state is symmetric. When a state has the form of symmetry corresponding to transformation *T*, we say that the state is *T*-symmetric.

Strictly speaking, we must also consider the case in which it is a set of states rather than a single state that is symmetric. A set $\Sigma$ of states will have a given form of symmetry just in case $\Sigma =T\Sigma =\left\{ Ta:a\in \Sigma \right\}$, where *T* is the transformation corresponding to that form of symmetry. However, we will tacitly treat a set of multiple states as a single conjunctive state except in the few cases where the distinction matters.

Two aspects of the symmetry–invariance link should be emphasized. First, it concerns symmetry as a property of states, not as a property of objects. Of course, there *is *a sense in which an object may be symmetric or asymmetric. And it is an interesting question what exactly the relationship is between the object-targeting notion and the state-targeting notion. But it is not a question we will address here. Second, invariance in the symmetry–invariance link is a matter of numerical identity. The image of the state under the transformation must be numerically identical to the original state, not merely the same in certain respects.

To see the symmetry–invariance link in action, imagine a chessboard at the start of a game. Consider the spatial distribution of black and white squares: here black, here white, here black again, and so on. It has twofold or 180^∘^ rotational symmetry. This form of symmetry corresponds to the transformation ‘rotate by a half turn’. And indeed, if we take this distribution and rotate it by a half turn, we have the same distribution again. The distribution is invariant under the corresponding transformation.

States which are closely related to one another may nonetheless differ with respect to symmetry. For example, although the spatial distribution of black and white squares has twofold rotational symmetry, this is not the case for the spatial distribution of black and white squares *and* chess pieces. Since the pieces on opposite sides of the board are of opposing colors, this distribution lacks twofold rotational symmetry. And indeed, it is not invariant under rotation by a half turn.

Every form of symmetry corresponds to a transformation, but not every transformation corresponds to a form of symmetry. For example, given an arbitrary state *a*, *a* is the only state that is invariant under the transformation that maps every state to *a*. Yet that hardly shows that there is a form of symmetry uniquely possessed by *a*. It is therefore only certain transformations invariance under which makes for symmetry. Which transformations are those? For present purposes we can leave this difficult question unanswered.

Having made these clarifications, we can now offer our final statement of the symmetry principle.

*No Asymmetry from Symmetry* In a full, unpruned grounding explanation, if the explanans has a given form of symmetry, the explanandum will have that same form of symmetry.

### The principle defended

We will adopt a conception of grounding explanation on which such explanations have three parts (as in (Glazier, 2016) or (Schaffer, 2017)). There is the explanandum, or what gets explained; the explanans, or what does the explaining; and a ‘metaphysical law’ governing the way in which the explanans gives rise to the explanandum. To give a simple example, we might offer a grounding explanation of why a certain barn is red by appealing to the fact that it is crimson together with a metaphysical law to the effect that whatever is crimson must also be red. The law governs the way in which, in general, the facts about which things are crimson ground the facts about which things are red.^4^

Perhaps there can be grounding explanations in which the explanans and explanandum are connected, not by a law, but in some other way, such by the obtaining of a certain special kind of determination relation. However, I will make the plausible assumption that there is always some general law under which such particular connections are subsumed, a law which governs the way in which, in general, facts like the explanans ground facts like the explanandum. (I do not assume that the law must be in any sense fundamental or basic.) And so I will assume that whenever one fact provides a grounding explanation of another, a grounding explanation is always available in which the two are connected by a law. I will therefore confine my discussion to such law-involving explanations.

With each metaphysical law we may associate a function *L* that maps each state *a* to the (unpruned) result of applying the law to *a*, which we assume is defined not only for actual states but for possible states as well. In view of this association we may (and will in what follows) move easily between talk of laws and of their associated functions.^5^

Let us say that *T* is a *symmetry transformation* if it corresponds to a form of symmetry in the way described in Sect. 2.1. Such transformations interact with metaphysical laws in a quite distinctive way. To see what I mean, imagine a tile mosaic depicting various familiar objects arranged in no particular manner: tables, chairs, lamps, and so on. The spatial distribution of these images of familiar objects admits of grounding explanation in terms of the spatial distribution of the tiles. But now imagine we take the whole mosaic and rotate it clockwise by a quarter turn. What will happen to our images of familiar objects? Well, nothing—or rather, nothing except that they too will rotate clockwise by a quarter turn. In every other respect, they will remain the same.

Let us put the point in more abstract and general terms. Let *a* be the state of the spatial distribution of the (unrotated) tiles, let *L* be the law governing how, in general, facts about mosaic tiles give rise to facts about the images those tiles constitute, and let *b* be the result of applying *L* to *a*. Now let *T* be the transformation ‘rotate clockwise by a quarter turn’. Since rotating *a* has no effect on *b* beyond rotating *it* in the same way, we know that if we rotate *a*, and then apply the law, the result will be identical to the result of simply rotating *b*. In symbols: $LTa=Tb$. Since $b=La$, by substitution we obtain $LTa=TLa$.

Of course, there is nothing special about *a* in particular. No matter what state *a* is, we will have $LTa=TLa$—and so in general the law *L* commutes with the symmetry transformation *T*.

The phenomenon of commutativity is not confined to tile mosaics and images. Think of an explanation of the location of a table in terms of the arrangement of its legs and top, of an explanation of a temperature distribution in terms of a distribution of molecular kinetic energy, of an explanation (supposing one exists) of the distribution of beauty in a museum in terms of the distribution of paint on canvas, and so on. In any case in which a state *a* provides a grounding explanation of a state *b* in accordance with a law *L*, rotating *a* will do nothing to *b* beyond rotating it too. And so, just as above, we will have $LTa=TLa$.

Nor is the phenomenon confined to rotation. It appears to arise for any symmetry transformation. Thus a spatial or temporal translation of *a* will do nothing to *b* beyond translating it too. Similarly, a spatial reflection of *a* will do nothing to *b* beyond reflecting it. If, for instance, the state of the arrangement of fibers in this right-handed glove is reflected in the obvious way, nothing will happen to the glove’s location except that it too will be reflected, transforming from right-handed to left-handed.

In some contexts, to be sure, one does encounter so-called symmetry transformations which do not commute with the metaphysical laws. For instance, in mathematics any one-to-one mapping of a set onto itself is sometimes called a symmetry of that set.^6^ This suggests a broad conception on which any permutation of the objects involved in a state will count as a symmetry transformation. So understood, not every symmetry transformation will commute with the laws.

Suppose, for example, that some dots are arranged in a row from left to right, and suppose further that they are arranged in two groups, with the left group of dots being all red and the right group being all blue. Take the state of the dots’ arrangement and apply the law governing how, in general, facts about discrete objects give rise to facts about the groups constituted by those objects. The result is, roughly, the state of a red group being on the left and a blue group being on the right. Now apply to that resulting state the permutation transformation ‘swap the leftmost element and the rightmost element’. The effect is to swap the two uniformly colored groups, ending up with the state of a blue group’s being on the left and a red group’s being on the right. But if, instead, one first permutes and then applies the law, the result is different. The permutation transformation swaps the leftmost red dot for the rightmost blue dot while leaving the others unchanged. And so, if we now apply the law, the result is not one blue group and one red group but rather two variegated groups. Commutativity fails.

However, the proper response to this case is not to abandon the claim that symmetry transformations commute with metaphysical laws, but rather to insist that permuting the leftmost and rightmost elements of an array is not a genuine symmetry transformation. There is no reason to think that a state which is invariant under this left-right permutation will be symmetric in any intuitive sense. (Of course, this is not to deny that it may be useful for mathematical purposes to regard it as symmetric.)

Having argued that metaphysical laws commute with symmetry transformations, we are now in a position to offer a proof of our symmetry principle, which, for convenience, we restate^7^

*No Asymmetry from Symmetry* In a full, unpruned grounding explanation, if the explanans has a given form of symmetry, the explanandum will have that same form of symmetry.

#### Proof

Suppose we have a full, unpruned grounding explanation involving a *T*-symmetric explanans *a* and a law *L*. The explanandum of this explanation is then *La*. By the symmetry–invariance link we have $a=Ta$ and so $La=LTa$. Since *L* commutes with *T*, this yields $La=TLa$, and so by the symmetry–invariance link, *La* is *T*-symmetric.

### Symmetry and invariance

As foreshadowed, Sect. 2.1’s statement of the symmetry–invariance link requires revision. The cases that bring out the need for revision may seem recherché, and it is tempting to simply dismiss them. But a proper treatment of these cases turns out to be crucial for the objections to be developed in Sect. 3.

Here is the simplest such case. Imagine a world in which time has a beginning but nothing ever exists and nothing ever happens. God, if you like, creates an empty world and then straightaway rests—forever. Let the *history* of this world be the total state of time and of what occurs in time. Then intuitively, this world’s history lacks temporal translational symmetry. After all, it has a temporal ‘edge’, a first moment, which is present once and then never again. Because this history lacks temporal translational symmetry, the symmetry–invariance link requires it to be non-invariant under the transformation corresponding to that form of symmetry, namely translation in time.

That last sentence is not quite correct. There is no single transformation corresponding to temporal translational symmetry. For ‘temporal translational symmetry’ is not a fully specific form of symmetry. There are many forms of temporal translational symmetry, each corresponding to a translation of a particular temporal distance and direction. Thus there is a form of translational symmetry corresponding to translation backward by one year, to translation forward by five years, by ten years, and so on. But the history of our imagined world lacks *any* of these forms of temporal translational symmetry. So let us pick some translation, say translation forward by *d* years. Then the symmetry–invariance link requires the history to be non-invariant under this transformation.

But is this requirement met? It depends on how the translation transformation is understood. There is a temptation in this case to understand the transformation as operating on only part of the state it is given—and in particular, to take it to be a translation only of *what occurs in time* and not of *time itself*. But our imagined world’s history will be invariant under such a translation. The ‘contents’ of time are void and so translating them leaves everything as it is. And so the symmetry–invariance link will entail, incorrectly, that our imagined world’s history is translationally symmetric.

In order for the symmetry–invariance link to accommodate this case, we must resist this temptation. We should insist on the—-upon reflection, sensible—requirement that the transformation should operate on the entirety of the state it is given. In the present case, ‘the state it is given’ is the imagined world’s history: the total state of time and its contents. The upshot is that not only the contents of time but time itself must be translated. (Of course, if in some *other* case, the transformation is given a state that does not involve time itself but only its contents, then only those contents should be translated.)

But the problem now is how to understand the idea of translating time itself. What could it be translated with respect to?

Let us recognize an operation of *embedding* a given state in a fixed, ‘reference’ spatiotemporal coordinate system.^8^ I will not attempt to characterize this operation in full generality but will instead confine myself to the case in which the ‘input’ state involves only material objects, events, points and regions of space, and moments and intervals of time. (This suffices for our purposes.) The operation maps this input state to another state, called the embedding, which augments the input state with an assignment of coordinates—*n*-tuples of numbers—to its objects, events, points, regions, moments, and intervals.

We are now able to give our final statement of the symmetry–invariance link.

*Symmetry–invariance link* The state *a* has a given form of symmetry just in case its embedding is invariant under the corresponding transformation *T*.

 With this revision, our imagined empty world becomes translationally asymmetric, just as we desired. To see this, begin by embedding that world’s history in a reference coordinate system, assigning a coordinate to, among other things, the first moment of time $t_{0}$. Now translate this embedding (i.e. translate it forward by *d* years). Since the reference coordinate system is fixed, the result is a state in which the contents of time, as well as time itself, are shifted with respect to that coordinate system. And so $t_{0}$ will have a different coordinate than it had before the translation. Thus the embedding is not invariant under the translation transformation, and so the revised link entails, correctly, that this world’s history is asymmetric.

In motivating the translational asymmetry of our imagined world’s history, we stressed the fact that time in that world has a first moment. But although this is a particularly vivid source of asymmetry, the history of this world in fact contains infinitely many such sources, one for each moment of time. To see this, let *t* be an arbitrary moment. Like $t_{0}$, *t* is present once and then never again. And indeed, if we embed this history in a reference coordinate system, and then translate that history with respect to it, the coordinate of *t* will change. The mere fact that the history contains a nonrepeating sequence of distinct moments ensures, all on its own, its temporal translational asymmetry. Even an empty world in which time has no beginning and no end will have an asymmetric history.

Symmetry, evidently, is a distinction not easily attained. But nor is it impossible. We have already seen several examples of symmetric states, and we will see several more below.

Our formulation of the symmetry–invariance link has given an important role to coordinate systems. But important though such systems are, they need not be seen as real. They may—and in my judgment, should—be viewed as a mere ‘heuristic’ convenience: very useful for inferring from invariance to symmetry or vice versa, far less so for giving a metaphysically serious account of reality.

We commonly adopt such an attitude toward the use of coordinate systems in science. For example, we may embed a comet in a coordinate system in order to calculate its trajectory, but we do not think this requires us to view the coordinates we assign to the comet as being real in the way the comet is. Of course, to observe that this attitude is common is not to deny that it gives rise to philosophical concerns. If coordinate systems are somehow unreal then we must eventually account for whatever calculational practices presuppose them in terms that do not presuppose them. But this is a large task and must be left for another time.^9^

The role of coordinate systems in the symmetry–invariance link *would* be disturbing if we took the link to be somehow definitional of or essential to symmetry. For surely the symmetry of the state of a material object, say a honeycomb, does not essentially involve abstract objects like coordinates. But we assume only that the symmetry–invariance link obtains, not that it gives symmetry’s essence. The question of what exactly that essence is we also leave for another time.

Our proof of the symmetry principle in Sect. 2.2 appealed to the unrevised symmetry–invariance link. But it can be modified to accommodate the revised link given one assumption. Let *L* be a law, *a* a state, and *E* the function that takes any state to its embedding. We assume that $ELa=LEa$ on the grounds that whether a state includes a reference coordinate system should make no difference to the operation of the law. It should not matter whether we first apply the law to a state and then embed the result in a coordinate system, or whether we first embed the state and then apply the law. The law, so to speak, ignores the coordinate system altogether.

Here, then, is our modified proof of the symmetry principle.

#### Proof

Suppose we have a full, unpruned grounding explanation involving a *T*-symmetric explanans *a* and a law *L*. The explanandum of this explanation is then *La*. By the assumption just made, we have $ELa=LEa$, from which it follows that $TLEa=TELa$. Since *L* commutes with *T*, we have $LTEa=TLEa$. And since *a* is *T*-symmetric, by the revised symmetry–invariance link we have $Ea=TEa$, from which it follows that $LEa=LTEa$. Chaining identities we obtain $ELa=TELa$, and so by the revised symmetry–invariance link, *La* is *T*-symmetric.

## The objections

I turn now to the objections posed by the symmetry principle to the four reductionist views listed in Sect. 1: qualitativism, relationalism, the tenseless theory of time, and comparativism. The details of these views mean that the objections must take a somewhat different form in each case and so it is necessary to develop each individually. But although the objections exhibit some diversity they also possess an evident unity. Their similarity is a testament to the power and scope of the symmetry principle.

### Qualitativism

We may distinguish facts that concern particular individuals from facts that do not. The former are *individualistic*, the latter *qualitative*. For example, the fact that Socrates is a philosopher is individualistic, while the fact that there are philosophers is qualitative.

In 2014, Shamik Dasgupta posed the following question:

Now, of the qualitative and the individualistic, which are the more fundamental? A natural view is that the most fundamental facts are individualistic facts about how a domain of individuals are propertied and related to one another, and that they are sufficient to ground ... the qualitative facts. Let us call this *individualism*. In contrast, let *qualitativism* be the opposite view that the most fundamental facts are qualitative facts and that they are sufficient to ground ... the individualistic facts. (6)

Although Dasgupta acknowledges that ‘individualism is perhaps the more natural position’ (6), he himself favors qualitativism on the grounds that

if individualism were true then the individualistic facts of our world would lie beyond our epistemic ken. The idea is that our knowledge of the world is limited to knowledge of its qualitative nature and whatever is grounded in that qualitative nature, and since individualism implies that there are further facts of the matter as to which particular individuals lie behind those qualities it follows that those facts would be unknowable. (6)

But whatever epistemic concerns may be raised by individualism, its qualitativist rival faces a powerful objection.^10^ The objection is that qualitativism leads to violations of the no-asymmetry-from-symmetry principle.

To see why, consider a ‘frieze world’ whose material contents repeat infinitely along a left–right axis, in both directions, every *d* light years. To fix ideas, suppose that at point *p* there is a blue planet; call it ‘Terra’. Then there will be infinitely many other blue planets spaced evenly along the axis in both directions from *p*.

The qualitative facts in this world possess a certain translational symmetry. The frieze-like character of the world makes this symmetry intuitively clear, but it can be further supported by appeal to the symmetry–invariance link. The qualitative facts taken together (strictly speaking, their embedding—see Sect. 2.3) are invariant under the transformation ‘translate rightward by *d* light years’. Before the transformation, for example, we have a blue planet at coordinates $\langle p_{n}\rangle$, another qualitatively identical blue planet *d* light years to the right of $\langle p_{n}\rangle$, another such planet *d* light years to the left of $\langle p_{n}\rangle$, and so on. And after the transformation, we have exactly the same qualitative facts (strictly speaking, the same embedding of qualitative facts—I will mostly leave such qualifications tacit from now on). They are invariant under the transformation and are therefore translationally symmetric.

But the individualistic facts are not. Again, this is intuitively clear. For although the world qualitatively repeats as one moves along the axis, it does not individualistically repeat. There are infinitely many planets just like Terra, but there is only one Terra.

This claim of asymmetry may be further supported by appeal to the symmetry–invariance link. Before the transformation, we may suppose, Terra is at coordinates $\langle p_{n}\rangle$. After the transformation, however, Terra is *d* light years to the right of $\langle p_{n}\rangle$. The individualistic facts are not invariant under the transformation and are therefore translationally asymmetric.

But the qualitativist holds that the individualistic facts are grounded in the qualitative facts.^11^ The former therefore have a grounding explanation in terms of the latter. Yet the former are asymmetric while the latter are symmetric. We therefore have a violation of the no-asymmetry-from-symmetry principle.

That is the essence of the objection. But if it is to be sustained two details require care.

The first concerns the adicity of the grounding relation,^12^ I said that the qualitativist holds that the (asymmetric) individualistic facts are grounded in the (symmetric) qualitative facts: a violation of the symmetry principle. But on the standard view ‘grounded in’ is one-many: a *single* fact is grounded in one or more others.^13^ The qualitativist is therefore more perspicuously described as holding that *each* individualistic fact is grounded in one or more qualitative facts. But then can we be sure she is really committed to a violation of the symmetry principle?

Yes. To see why, call the conjunction of all individualistic facts (save that very conjunction itself) the *global individualistic state*. That big conjunction is itself a single fact, one that is asymmetric for the same reason the individualistic facts taken together are. And we can show that the qualitativist is committed to the claim that the (asymmetric) global individualistic state is grounded in the (symmetric) qualitative facts taken together.^14^ The argument for this has three steps.

First, given the standard assumption that a conjunctive fact is grounded in its conjuncts taken together, the global individualistic state is grounded in the totality of the individualistic facts taken together (with the exception of the global individualistic state itself—I will leave such qualifications tacit from now on).

Second, the qualitativist is committed to taking the totality of individualistic facts to be *distributively grounded*, in Kit Fine’s (2012a: 54) sense, in the totality of qualitative facts. I give a proof of this claim, highlighting one detail that will be especially important in Sect. 3.2.

#### Proof

The set of facts $\Delta$ is said to be a distributive ground for the set of facts $\Gamma$ iff for some $\Delta _{1},\Delta _{2},\ldots$ such that $\Delta =\Delta _{1}\cup \Delta _{2}\cup \ldots$ and for some $C_{1},C_{2},\ldots$ such that $\Gamma =\left\{ C_{1},C_{2},\ldots \right\}$, the members of $\Delta _{1}$ taken together ground $C_{1}$, the members of $\Delta _{2}$ taken together ground $C_{2}$, and so on. Let *Q* be the set of qualitative facts, and let *I* be the set of individualistic facts. To see that the qualitativist will take *Q* to be a distributive ground for *I*, notice first that (this is the important detail) any qualitative fact $Q_{j}$ will ground some individualistic fact. For instance, $Q_{j}$ will ground the fact that $Q_{j}$ exists or obtains; call this latter fact $I_{j}$. (The fact $I_{j}$ is, of course, distinct from the fact $Q_{j}$.) Now let $I^{*}$ be the set of all $I_{j}$. Then since $Q=\left\{ Q_{1}\right\} \cup \left\{ Q_{2}\right\} \cup \ldots$, $I^{*}=\left\{ I_{1},I_{2},\ldots \right\}$, and $Q_{j}$ grounds $I_{j}$ for all *j*, *Q* is a distributive ground for $I^{*}$. But the qualitativist holds that each of the remaining individualistic facts (those not in $I^{*}$) is grounded in one or more qualitative facts and thus in some subset of *Q*. So she will take *Q* to be a distributive ground not only for $I^{*}$ but for *I* as well.

Third, we chain together the first two steps to conclude that the qualitativist must take the global individualistic state to be grounded in the totality of qualitative facts taken together.^15^ She must therefore take there to be a grounding explanation of the former in terms of the latter. Yet the former is asymmetric while the latter are symmetric: a violation of the symmetry principle.

The second detail which requires care concerns the explanandum of this symmetry-principle-violating explanation. We have seen that the symmetry principle applies only to unpruned explanations. And the qualitativist’s explanation of the global individualistic state is perhaps not unpruned. There *might* be further facts that are also explained by the qualitative facts together with the the purported metaphysical law to which the qualitativist’s grounding explanation appeals, the law which she takes to govern the general way in which qualitative facts give rise to individualistic facts. But whatever these further facts might be, it is hard to see how by adding them to the qualitativist’s explanation we would transform its asymmetric explanandum into a symmetric one. Only one of the infinitely many blue planets is Terra. That is where the asymmetry comes from, and it is hard to see how the addition of any further facts could change that.^16^

I conclude that the qualitativist is committed to the claim that in the frieze world, a symmetric explanans provides a full, unpruned explanation of an asymmetric explanandum. But this is precisely what the symmetry principle proscribes. If that principle is correct, qualitativism cannot be.

This objection to qualitativism could equally (if less straightforwardly) be made by appeal not to the frieze world but to the well-known ‘Max Black’ world, whose sole material occupants are two qualitatively identical iron spheres *A* and *B* separated by a distance of two miles. In the Max Black world, the qualitative facts have reflection symmetry: reflect that state across the plane which intersects the midpoint of the line between the spheres’ centers and is orthogonal to that line, and you have the same state back again. But the global individualistic state does not have reflection symmetry, since reflecting *that* state swaps the locations of *A* and *B*. And yet the qualitativist must say that the qualitative facts taken together will provide a grounding explanation of the global individualistic state. This leads to a violation of the symmetry principle.

Dasgupta’s version of qualitativism differs subtly from the one discussed so far. *We* formulated qualitativism in terms of the standard one-many relation ‘is grounded in’: each individualistic fact is grounded in one or more qualitative facts. Dasgupta, by contrast, argues that the qualitativist should instead formulate the view in terms of an irreducible *many-many* relation ‘are grounded in’, and he considers a qualitativist view on which the individualistic facts (all of them) are grounded in (all of) the qualitative facts.

Dasgupta’s pluralist qualitativism, like the standard, non-pluralist version, faces the objection from the symmetry principle. Indeed, the objection is easier to mount against pluralist qualitativism than it is against the standard, non-pluralist version. For there is no longer any need to carefully identify a single fact (the global individualistic state) which is grounded in the totality of qualitative facts. The claim that the individualistic facts are grounded in the qualitative facts will be perfectly acceptable as it stands. Yet in the frieze world (and in the Max Black world) it leads to violations of the symmetry principle.^17^

We have in this section developed the objection to qualitativism from the symmetry principle in precise terms, and we will do the same with the other objections. But the objection, and indeed the principle itself, possess an intuitive force independent of any precise formulation. Consider again the frieze world. The translational symmetry of the qualitative facts in that world can be seen as a form of sameness. The frieze world—or the ‘qualitative aspect’ of that world—is the same at this location as it is at that location. How then can it give rise to an individualistic world that is *not* the same? How can sameness become difference in this way? The plausibility of the no-asymmetry-from-symmetry principle reflects our intuition that it cannot.

### Relationalism

Let us take relationalism to be the view that each fact about space (i.e. about points or regions of space) is grounded in one or more facts about the spatial relations between material bodies. For example, it is a fact that the region of space inside the Kaaba in Mecca is roughly cubical, and a relationalist might take this fact to be grounded in the fact that the Kaaba itself is roughly cubical. Relationalism, I will argue, faces an objection from the no-asymmetry-from-symmetry principle.

The form of relationalism just defined is a prominent one. But as I observed in Sect. 1, there are other forms too. There are eliminativist forms on which there are simply no facts at all about space itself, only facts about material bodies and the spatial relations between them. There are also non-eliminativist forms of relationalism which take the grounds for facts about space to be partly modal, involving not only facts about the actual spatial relations between bodies but facts about merely possible relations as well. I leave discussion of these alternative forms of relationalism for another day. I must also postpone the important question of how exactly the symmetry objection applies in a relativistic setting in which what is at issue are facts about spacetime rather than space. We have to crawl before we walk and a proper formulation of the objection in the non-relativistic setting is challenging enough.

The symmetry objection to relationalism is broadly similar to the objection to qualitativism, but it is also importantly different. To see why, recall that to mount the objection to qualitativism (assuming the standard one-many grounding relation) we had to identify a single individualistic fact which the qualitativist would have to take to be grounded in the totality of qualitative facts. And our argument that the qualitativist is committed to the existence of such a fact involved a proof which appealed to the claim that every qualitative fact grounds some individualistic fact. But the relationalist need not embrace the analogous claim that every relational fact grounds some spatial fact. For she might hold that it is only an elite subclass of the relational facts—facts about betweenness and congruence, perhaps—that ground the facts about space.

We will therefore develop the objection to relationalism in a somewhat different way. Consider a world consisting of a solitary motionless point particle *p*. Let $\theta$ be an arbitrary nontrivial angle (i.e. one not equivalent to 360^∘^); then the facts about the spatial relations between material bodies in this world are rotationally symmetric about *p* with respect to $\theta$ (in an arbitrary plane $\pi$). This is intuitively clear upon reflection on what these facts are. They include facts like ‘*p* is as far from *p* as *p* is from *p*’, ‘*p* is zero meters from *p*’, and ‘*p* lies between *p* and *p*’. The facts about such relations will be invariant under rotation by $\theta$ about *p*.

Not only are these relational facts symmetric when taken together, each one is symmetric on its own. The fact that *p* is as far from *p* as *p* is from *p*, for instance, is on its own invariant under rotation by $\theta$ about *p*, and the same is true of every other relational fact.

Consider now the facts about points and regions of space. Among them are facts about particular rays or half-lines whose initial point is *p*’s location. Let *R* be one such ray (in the plane $\pi$). It will point in some unique direction *D*. And this is already enough to intuitively show that the facts about space taken together are not rotationally symmetric about *p* with respect to $\theta$. For direction *D* is distinguished from all other directions by being the direction of *R*. Of course, there are other rays pointing in other directions, but *D* is the only direction that is *R*’s.

Further support for asymmetry is provided by the symmetry–invariance link. For the ray *R* is not invariant under rotation by $\theta$. It points in direction *D* before the rotation and in a different direction afterwards. Or more carefully, suppose we embed the facts about space in a coordinate system as in Sect. 2.3. Let $\langle r_{n}\rangle$ be the coordinates of some non-initial point in *R*. Now suppose we rotate the facts about space with respect to the coordinate system. After that rotation, *R* will no longer contain the point with coordinates $\langle r_{n}\rangle$. Thus taken together the embedding of the facts about space is not invariant under the rotation, and so those facts are rotationally asymmetric.

Now call the conjunction of all facts about space the *global spatial state*. This conjunctive fact will be grounded in its conjuncts, the spatial facts, taken together. And each of these conjuncts, according to the relationalist, will be grounded in some plurality of relational facts. Let *U* be the union of all such pluralities. Then the set of spatial facts, it may be shown, is distributively grounded in *U*. And so by the same chaining principle we appealed to in Sect. 3.1, the global spatial state will admit of grounding explanation in terms of the members of *U* taken together.

We argued above that the facts about space taken together are not rotationally symmetric, and so neither is the global spatial state. By contrast, *U* *is* rotationally symmetric. For *U* contains only facts about the spatial relations between material bodies. We argued above that each such fact is rotationally symmetric. Since each member of *U* is symmetric, so is *U* as a whole.

The relationalist must therefore say that a symmetric explanans provides a grounding explanation of the asymmetric global spatial state. And although this explanation is perhaps not unpruned, just as in Sect. 3.1 it seems that even if its explanandum were appropriately filled out, the resulting unpruned explanandum would still be asymmetric.

The relationalist is therefore committed to the claim that in this point-particle world, the no-asymmetry-from-symmetry principle is violated. If that principle is correct, then relationalism cannot be.

### The tenseless theory of time

Suppose we admit a distinction between tensed facts, like the fact that it is sunny, and tenseless facts, like the fact that April 9, 2024 is a sunny day. We may then consider the tenseless theory of time (also called the ‘B’ theory), the view that each tensed fact is grounded in one or more tenseless facts.

This is a nontraditional version of the tenseless theory. The traditional theory was a view, not about the grounds of tensed *facts*, but about the truth conditions of tensed *sentences*—namely, that these conditions are tenseless, or can be made to be so (see e.g. (Sider, 2001): 12–24). But in these heady days following what Jonathan Schaffer (2016: 91n) has called the ‘grounding revolution’, our nontraditional formulation is a natural one to consider—and one which philosophers are increasingly adopting.^18^

This formulation of the tenseless theory of time faces an objection from the no-asymmetry-from-symmetry principle. At least initially, the objection may be developed along the lines of Sect. 3.1’s objection to qualitativism.

Consider a world of two-way eternal recurrence, in which history repeats itself endlessly, with no first epoch and no last epoch. To fix ideas, suppose that in every epoch a new messiah is born.^19^ The tenseless facts in this world, one might think, are jointly translationally symmetric—they possess a certain temporal form of translational symmetry. For suppose we shift forward or ‘postpone’ them all by *d* years, where this is the duration of each epoch. This transformation may appear to leave the tenseless facts unchanged. Consider, for example, the messianic births. Although each birth will be postponed by *d* years, an earlier birth will take its place.

The tensed facts in this world, however, are *not* translationally symmetric in this way. They distinguish one moment above all others as being present, and so they are not invariant under the postponement transformation. (More carefully, suppose we embed the tensed facts in a coordinate system as in Sect. 2.3. Then before the transformation the moment which is present will be at some particular coordinate, and after the transformation the moment which is present will be *d* years later than that coordinate. I will leave these sorts of remarks tacit from now on.)

The tenseless theory of time, then, appears to require that the tenseless facts, which are jointly symmetric, provide a grounding explanation of the tensed facts, which are asymmetric (and presumably would remain so upon depruning). This threatens to violate the no-asymmetry-from-symmetry principle.

Now as before, we must take care to respect the adicity of ground. Strictly speaking, we must argue that the tenseless theorist is committed to taking some *single* asymmetric tensed fact to admit of grounding explanation in terms of the symmetric tensed facts. But this can be done. The single tensed fact we require is the conjunction of all tensed facts, what we may call the *global tensed state*. The details are much as they were in Sect. 3.1; I spell them out in a footnote.^20^

This argument against the tenseless theory of time will not work as it stands. The reason is that the tenseless facts in the world of eternal recurrence are not translationally symmetric. To see this, consider the messianic birth that occurs at *t*. This is the birth of a particular messiah; call him Brian. The tenseless facts then distinguish *t* above all other moments as being the moment at which Brian is born. There are other moments at which other messiahs are born, but *t* is the only moment which witnesses the birth of Brian. The tenseless facts are not invariant under postponement by *d* years, and they are not translationally symmetric.

The problem is this. In general, in a world of two-way eternal recurrence, the *qualitative* tenseless facts will be translationally symmetric, but the *individualistic* tenseless facts will not be. So in such a world, the *totality* of tenseless facts lacks symmetry. It is therefore consistent with the no-asymmetry-from-symmetry principle to take those asymmetric tenseless facts to ground the asymmetric global tensed state. And so our objection to the tenseless theory fails.

The objection can be salvaged, however, by appealing to a variant of the symmetry principle. Our original principle said: no asymmetry from symmetry. The variant says: no qualitative asymmetry from qualitative symmetry. As long as the qualitative part of the explanans is symmetric, the qualitative part of the explanandum will have to be symmetric as well.

This variant principle is scarcely less plausible than the original. To see why, let us return to the sunflower mosaic of Sect. 1, but let us describe the case differently than before. Let us *name* the mosaic’s tiles: $t_{1}$, $t_{2}$, $t_{3}$ and so on up to $t_{n}$. And let us also name the parts of the sunflower image those tiles constitute: *c* for the center and $p_{1},\ldots ,p_{m}$ for the petals. The arrangement of $t_{1},\ldots ,t_{n}$ is rotationally symmetric. It therefore follows, via a sort of reasoning that by now is familiar, that the arrangement of *c* together with $p_{1},\ldots ,p_{m}$ must be rotationally symmetric as well.

However, the no-asymmetry-from-symmetry principle as stated in Sect. 2.1 is doubly inapplicable here. That principle says that a symmetric explanans must give rise to a symmetric explanandum. But the explanans here is not strictly symmetric. For it involves the individual tiles $t_{1},\ldots ,t_{n}$, and since each tile appears at only one place within the mosaic, the arrangement of $t_{1},\ldots ,t_{n}$ is not strictly invariant under rotation (by any nontrivial angle). In the same way, the *explanandum* is also not strictly symmetric, since it involves the individual petals $p_{1},\ldots ,p_{m}$.

Still, our inference seems justified all the same. What supports the inference, I submit, is not the no-asymmetry-from-symmetry principle but its variant. Because the qualitative part of the explanans is symmetric, we may infer that the qualitative part of the explanandum will be symmetric too.

The plausibility of the variant may be further supported by appeal to the other cases discussed in Sect. 1. Consider the case of the origami crane. If the explanans involves the individual left and right halves $h_{1}$ and $h_{2}$ of the folded sheet of paper, then it is not reflectionally symmetric, and yet it seems clear that a no-asymmetry-from-symmetry inference is still justified. Or consider the decorative border. If the explanans involves the individual line segments that compose the border, then it is not translationally symmetric. Yet the inference is justified all the same. What supports all of these inferences is the variant principle.

But how exactly should we state this variant? The original no-asymmetry-from-symmetry principle was this:

In a full, unpruned grounding explanation, if the explanans has a given form of symmetry, the explanandum will have that same form of symmetry.

We may obtain the variant principle by a straightforward modification.

*No Asymmetry from Symmetry (variant)* In a full, unpruned grounding explanation, if the qualitative part of the explanans has a given form of symmetry, the qualitative part of the explanandum will have that same form of symmetry.

I have not tried to give a precise characterization of the distinction between individualistic and qualitative facts, and nor will I try to give a precise characterization of the notion of qualitative part. But the intuitive idea is that the qualitative part of a state *a* is a qualitative state that is as comprehensive as it can be without going beyond *a*. The qualitative part of the state of the solar system, for instance, will consist in there being eight planets, with certain physical characteristics, at certain distances from one another, etc. But it will not involve individuals like Earth or Mars.

We are now in a position to resurrect our objection to the tenseless theory of time. For the tenseless theorist, the tenseless facts provide a grounding explanation of the tensed facts (better: the global tensed state). In the world of eternal recurrence, the tenseless facts’ qualitative part has temporal translational symmetry (even if the tenseless facts themselves do not). The variant principle therefore requires the qualitative part of the global tensed state (once it is ‘depruned’) to be translationally symmetric. But it seems not to be, since it—qualitatively!—distinguishes one moment above all others as *present*. Thus if the variant principle is correct, the tenseless theory of time cannot be.^21^

### Comparativism

We may distinguish two classes of facts about mass. On the one hand, there are the facts about the intrinsic masses of material bodies, such as the fact that the mass of this electron is $m_{e}$ or the fact that the mass of my laptop is 1.25 kg. On the other hand, there are the facts about mass relationships, such as the fact that my old laptop is more massive than my current one or the fact that this proton is two thousand times as massive as this electron. The former we may call *absolute* facts, the latter *comparative*. A similar distinction may be drawn for facts about quantities other than mass.

Is one of these classes of fact more basic than the other? The *comparativist* about a given quantity takes the comparative facts to be more basic. She holds that the the absolute facts about that quantity are grounded in comparative facts about that quantity. The comparativist about mass, for instance, holds that the intrinsic mass facts are grounded in facts about mass relationships. The *absolutist* about a given quantity, by contrast, holds that there are absolute facts about that quantity that are not grounded in comparative facts (or in other non-absolute facts).

Dasgupta (2013) argues for comparativism on epistemic grounds similar to those he adduced in favor of qualitativism (Sect. 3.1).^22^ But as he recognizes, these considerations are not decisive. And comparativism, I will argue, faces the strong objection that it leads to violations of the variant principle of Sect. 3.3. I will argue for this conclusion in the case of mass, but the argument extends to the other quantities as well.

Begin by considering a world consisting solely of an array of equally-sized homogeneous balls separated by distance *d* and extending infinitely along the left-right axis in both directions. Each ball is twice as massive as the ball to its left. Thus if this ball here has a mass of 10 kg, then the ball to its left will have a mass of 5 kg and the ball to its right will have a mass of 20 kg.

The comparative mass facts in this world are translationally symmetric—or at least their qualitative part is. Like the qualitative facts of Sect. 3.1’s frieze world, the comparative facts’ qualitative part repeats endlessly along the left-right axis: here a ball flanked by one half its mass and one double its mass, here another such ball, here still another, and so on. The qualitative part is invariant under the transformation ‘translate rightward by distance *d*’.

But the absolute mass facts in this world are not translationally symmetric in this way, and nor is their qualitative part. For choose any one of the balls. It will have some particular intrinsic mass *m*, and indeed it will be the only ball of this mass. Thus the absolute mass facts will distinguish this ball qualitatively from all the others as being the only one of mass *m*. A translation rightward by *d* will leave the unique ball of mass *m* in a different place from where it was before.

Here, then, is an initial statement of the objection. The comparativist will have to say that, in the infinite-array world, the absolute mass facts provide a grounding explanation of the comparative mass facts. But the comparative facts’ qualitative part is symmetric, while the absolute mass facts’ qualitative part is asymmetric. This violates the variant principle.

As is familiar by now, care must be taken with the details. The objection must be officially cast not in terms of the plurality of absolute facts but in terms of the ‘global absolute state’.^23^ And we must assume that the qualitative part of that state will remain asymmetric upon ‘depruning’.

Moreover, as in Sect. 3.2’s argument against relationalism, there is no guarantee that the comparativist will hold that *every* comparative fact must play a role in grounding the absolute facts. She may think it is only a certain kind of comparative fact, such as a fact about mass ratios or mass orderings, that serves to ground absolute facts ((Dasgupta, 2013): 109). If so, then the absolute facts (better: the global absolute state) will not admit of grounding explanation in terms of the totality of comparative facts, for some of those facts will be irrelevant to explaining why the absolute facts are as they are. Instead, the absolute facts will admit of grounding explanation only in terms of an elite subclass of comparative facts.

But this point hardly weakens our objection at all. For in the infinite array world any reasonable ground for the absolute facts will have a translationally symmetric qualitative part. The facts about mass ratios, for example, will satisfy this condition, as will the facts about mass orderings. And as long as the ground, whatever it is, has a symmetric qualitative part, the comparativist will run afoul of the variant principle. If that principle is correct, comparativism cannot be.

## The fragmentalist response

Fragmentalism is the strange view that there exist, or obtain, facts which are in a certain sense incompatible with one another. Different versions of fragmentalism differ with respect to which facts are taken to be incompatible. The tense-theoretic fragmentalist, for instance, takes there to be incompatible tensed facts, while the first-personal fragmentalist takes there to be incompatible first-personal or egocentric facts.^24^

This paper has considered four views: qualitativism, relationalism, the tenseless theory of time, and comparativism. Each is a view about what is grounded in what. And each faces an objection from the no-asymmetry-from-symmetry principle (or its variant). This section develops a response to these objections.

According to this response, the objection in each case is to be overcome by adopting a fragmentalist view of the grounded facts. Thus the objection to qualitativism is to be overcome by ‘going fragmentalist’ about the individualistic facts, the objection to relationalism by going fragmentalist about the facts about space, the objection to the tenseless theory of time by going fragmentalist about the tensed facts, and the objection to comparativism by going fragmentalist about the absolute facts.

Of these four fragmentalist views, only the tense-theoretic one has received any attention from philosophers. I shall therefore focus my discussion on the case of the tenseless theory of time, since it is only there that tense-theoretic fragmentalism is relevant. The fragmentalist responses in the other three cases are analogous, but a full discussion must await another time.

The fragmentalist view of tensed facts is best understood by contrasting it with the standard view of such facts. We may distinguish the tensed facts that obtain at a given moment *t* from the tensed facts that obtain simpliciter. For example, let $t_{0}$ be the present moment and let $t_{1943}$ be a moment in 1943. At $t_{0}$, it is a tensed fact that Joe Biden is an adult. And at $t_{1943}$, it is a tensed fact that Biden is a child. On this much, the standard and fragmentalist views agree. But they disagree over which tensed facts obtain simpliciter. For the standard theorist, only the fact that Biden is an adult obtains simpliciter. The fact that Biden is a child, though it obtains at $t_{1943}$, does not obtain simpliciter. In general, the standard theorist holds that the only tensed facts which obtain simpliciter are those which obtain at the present moment.

For the fragmentalist, by contrast, both the fact that Biden is an adult as well as the fact that Biden is a child obtain simpliciter. And in general, if there is a moment *t* at which a given fact obtains, then no matter what *t* is—no matter whether it is past, present, or future—the fragmentalist will take the fact to obtain simpliciter. Because the facts that obtain at one time are, as a rule, incompatible with the facts that obtain at any other time, the fragmentalist holds that there are incompatible facts all of which obtain simpliciter. For example, the fact that Biden is an adult is incompatible with the fact that Biden is a child, and yet for the fragmentalist both facts obtain simpliciter. (Here we see the fundamental weirdness of fragmentalism.)

Return now to the objection of Sect. 3.3 to the tenseless theory of time. That theory, we argued, was in conflict with the no-asymmetry-from-symmetry principle (in its variant form). For we said that in the world of eternal recurrence, the tenseless theorist must take the tenseless facts, the qualitative part of which is translationally symmetric, to provide a grounding explanation of the tensed facts, the qualitative part of which is translationally asymmetric.

But *is* it asymmetric? The tenseless theorist can adopt either a standard or fragmentalist view of the tensed facts. If she adopts the standard view, then the qualitative part of the tensed facts is indeed asymmetric, for it entails the existence of a distinguished present moment. But once she goes fragmentalist, the qualitative part of the tensed facts becomes *symmetric*. For she will now maintain that if a given fact obtains at *t*, then no matter what *t* is, that fact will obtain simpliciter. Now whichever *t* we choose, at *t* there obtains the fact that *t* is present. The fragmentalist will therefore conclude that, for all *t*, the fact that *t* is present obtains simpliciter. The qualitative part of the tensed facts, then, no longer entails the existence of a distinguished present moment. And so there is no longer any conflict with the symmetry principle (or its variant—I will suppress such qualifications from now on).

We might think of the difficulty the symmetry principle poses for the standard version of the tenseless theory of time, and the fragmentalist response to this difficulty, in the following way. With respect to which facts obtain simpliciter, the standard theorist recognizes only one maximal internally compatible ‘fragment’ of tensed facts, comprising just those tensed facts that obtain at the present moment $t_{0}$. For her, this fragment is the only one there is. And in the world of eternal recurrence, although the tenseless facts are symmetric, this $t_{0}$-fragment is asymmetric. Since, for the standard theorist, the $t_{0}$-fragment comprises the totality of tensed facts, she must countenance asymmetry from symmetry in violation of the principle.

Now the fragmentalist does not deny that the $t_{0}$-fragment is asymmetric. What she denies is that it comprises the totality of tensed facts. For her, there are many fragments, each of which corresponds to a different moment and each of which obtains simpliciter. Each fragment, to be sure, is asymmetric: *within* the fragment, one moment is distinguished as present. But taken together, the totality of fragments, and so the totality of tensed facts, is symmetric. The fragments are like the petals of a sunflower: individually asymmetric but jointly symmetric. Thus the symmetry of the tenseless explanans is preserved in the tensed explanandum, just as the no-asymmetry-from-symmetry principle requires.^25^

This view, which combines fragmentalism and the tenseless theory of time, is an unfamiliar one.^26^ Philosophers have tended instead to combine fragmentalism with* tensed* theories of time— theories on which there are at least some tensed facts without a wholly tenseless ground. But we have argued that despite its unfamiliarity, the present view offers the tenseless theorist a response to the challenge posed by the symmetry principle.

Fragmentalism, however, is such a strange view that there is a real worry as to whether this response can be sustained. I cannot here offer a full appraisal of fragmentalism and I myself do not know whether it should in the end be accepted—by the tenseless theorist or by anyone else. But I do wish to point out two ways in which, in the present context, the view is more defensible than earlier discussions may suggest.

The first involves a flatfooted objection to incompatible facts. The tense-theoretic fragmentalist thinks that the fact that Biden is an adult and the fact that Biden is a child both obtain (simpliciter). But these facts are in a clear sense incompatible with each other, and one might flatfootedly reject the whole idea that there can be two facts which, though incompatible, both obtain.

In response, fragmentalists have appealed to a notion of *coherence*. They have said that some facts cohere with one another and others do not. And they have conceded that two facts which are incompatible with each other *and *which cohere with each other cannot both obtain. But they have insisted that when two incompatible facts do not cohere with each other, then both *can* obtain. So, for example, with regard to the fact that Biden is an adult and the fact that Biden is a child, the strategy will be to hold that these facts do not cohere with each other and to insist on these grounds that, despite their incompatibility, both facts may obtain.

But this response is only as clear as the notion of coherence. What is it for two facts to cohere with each other? Although fragmentalists have tended to resist answering this question, opting instead to take coherence as primitive, this invites the question ofwhy a lack of coherence should make it unproblematic for incompatible facts to obtain. It is not at all clear what the fragmentalist can say in response.

However, in the present context the notion of coherence can actually be defined. The tenseless theorist can say that for two tensed facts to cohere is simply for them to obtain at the same moment of time. If she adopts this definition of coherence, then her response to the flatfooted objection amounts to the suggestion that the weirdness of admitting incompatible facts vanishes if these facts obtain at different moments. Thus she will say that, since the fact that Biden is an adult obtains at $t_{0}$, while the fact that Biden is a child obtains at $t_{1943}$, there is no problem in taking both facts to also obtain simpliciter. I am not sure this response succeeds in entirely countering the force of the flatfooted objection, but it does seem to go at least some way toward making the incompatibility tolerable.

A second objection to fragmentalism targets, not the *obtaining* of incompatible facts, but their status as fundamental or metaphysically basic. Some philosophers have developed fragmentalist views on which incompatibility appears at the most basic level of reality. Fine (2005), for instance, writes:

One naturally assumes that in a correct account of reality all apparent contradictions will be ironed out. If something is both hot and cold, it must be because one part is hot and the other cold, or because it is hot and cold at different times, or because being hot is somehow compatible with being cold. But on the [fragmentalist] view, this fundamental assumption is given up. It is taken to lie in the character of reality that certain apparently contradictory aspects of it cannot be explained away. Reality may be *irredeemably* incoherent. (280–1)

But the tenseless theorist can embrace fragmentalism without abandoning Fine’s ‘fundamental assumption’. On her view, although the tensed facts are mutually incompatible, they are grounded in the tenseless facts, which are mutually compatible. Reality for her is indeed incoherent, but not irredeemably so.

I conclude that the fragmentalist version of the tenseless theory of time not only avoids any conflict with the no-asymmetry-from-symmetry principle but is also more defensible than one might have thought. This conclusion is not without a certain irony. Tenseless theorists have tended to regard it as an advantage of their view that it need not venture anywhere near the dark waters of fragmentalism. They have taken their position to be immune to the arguments for fragmentalism advanced by philosophers like Fine, which target only the *tensed* theorist. But if I am right, it may be the tenseless theorist who needs fragmentalism the most. For it promises to protect her from the threat posed by the symmetry principle.

## Conclusion

I have defended the principle that an asymmetry cannot hold in virtue of a symmetry, and I have argued that this principle supports strong objections to four reductionist views: qualitativism, relationalism, the tenseless theory of time, and comparativism. These applications demonstrate not only the principle’s power but also its broad utility for metaphysics. Although the objections can be avoided by embracing fragmentalism, it is unclear whether the resulting views can be sustained, and if they cannot, then I believe the objections are fatal.

The injunction ‘no asymmetry from symmetry’ may strike the enthusiastic metaphysician as overbearing and oppressive. It functions as a constraint on our theorizing, requiring us to reject otherwise attractive views which run afoul of it. But constraints are not a bad thing—provided they are well-justified. In metaphysics, perhaps more than in any other branch of inquiry, we need every constraint of *that* kind we can get.^27^

## Footnotes

1 I am grateful to Asya Passinsky for this example.

2 Much recent work has adopted this sort of non-eliminativist characterization of relationalism, including Schaffer (2009: 363), Dasgupta (2011; 2015) and North (2018).

3 An example from chemistry of this way of thinking about symmetry is found in Shriver et al. (2014: 188): ‘Symmetry operations are actions that leave the molecule apparently unchanged.’ For an example from mathematics, see Whitehead (1988: 198): ‘Given a polyhedron (such as a cube, for example), any rotation about an axis in space which transforms the polyhedron into itself is called a symmetry of the polyhedron.’

4 This talk of ‘governing’ can, but need not, be taken with metaphysical seriousness. One *might* adopt an ‘anti-Humean’ view of metaphysical laws, on which they genuinely govern in the sense of playing an active role in making certain facts obtain, but one might also adopt a ‘Humean’ view, on which the laws are mere summaries of independently existing regularities in the facts. On this issue, as on other issues concerning the precise nature of metaphysical laws, we may remain neutral. My own account of laws is given in Glazier (2016); other discussions include Rosen (2006), Sider (2011, pp. 274–278), Wilsch (2015a, b) and Schaffer (2017).

5 I am assuming here that there is such a thing as *the* result of applying a given law to a given state, even when that state is merely possible. Although this assumption is controversial ((Wasserman, 2015)), it is made plausible by the widely held (see Skiles 2020 for references) principles of Necessitation (necessarily, if *a* grounds *b*, then necessarily, if *a* obtains, *b* obtains) and Internalism (necessarily, if *a* grounds *b*, then necessarily, if *a* and *b* obtain, then *a* grounds *b*). Necessitation and Internalism together entail that necessarily, if *a* grounds *b*, then necessarily, if *a* obtains, *a* grounds *b*. And this strongly suggests that the general laws governing grounding work the same way across modal space and so the result of applying a given law to a given state will be the same in every possible world.

6 See, for example, Rosenberg (2022: 47): ‘Let $X\subseteq S$ be two sets. Then a symmetry of *X* with respect to *S* is a bijective function $f:S\rightarrow S$ such that $f(X)=X$.’

7 An important historical antecedent of the symmetry principle is due to Curie (1894: 401), who suggested that ‘when certain effects show a certain asymmetry, this asymmetry must be found in the causes which gave rise to them’ (trans. (Ismael, 1997: 168). The proof in the text is analogous to Earman’s (2004: 175–176) proof of a version of Curie’s principle.

8 There are in fact infinitely many embedding operations, one for each coordinate system. But any one of them will do.

9 Field (1980) is a well-known attempt along these lines.

10 Of course, these are not the only possible views. For instance, one might hold a mixed view on which some fundamental facts are individualistic and some qualitative. The present section is concerned to argue only that qualitativism is false, not that individualism is true.

11 I assume that qualitativism is necessarily true if true at all (and similarly for the other three views to be discussed below) and so that it is true in the frieze world.

12 I speak of the grounding *relation* but this is only for ease of presentation. The argument could equally well proceed in terms of the grounding *operation*. See Fine (2012a: 46–47) for discussion of the contrast between the operational and relational views of ground.

13 Dasgupta himself rejects the standard view; we consider his view below.

14 It is worth mentioning an alternative line of argument on which we adopt a less formal characterization of the global individualistic state, understanding it simply as a state which specifies everything about the way the individuals in the world are. The qualitativist is then plausibly committed to the claim that this state is grounded in the qualitative facts taken together. This alternative may appeal to those who worry that there can be no conjunction of all individualistic facts for Cantorian reasons (compare Grim’s 1984 argument that there is no set of all truths). I am grateful to Fabrice Correia for discussion of this issue.

15 This is an application of the Cut rule in deRosset’s (2014) logic of strict ground. This rule, as deRosset notes, is derivable in Fine’s (2012b) pure logic of ground.

16 Though perhaps not impossible: see Sect. 4, especially n. 24.

17 I therefore do not think Dasgupta is correct to say that ‘the qualitativist may concede the possibility of Max Black worlds and yet deny that they are problematic for her view’ (26).

18 It is adopted by Amijee (2021) and Werner (2021), for example, and perhaps also by Skow (2018: 1822). A related formulation is adopted by Cusbert and Miller (2018), who take the tenseless theory to be the view that the facts about what is past, present and future are grounded in the facts about what is earlier than what (or what is later than or simultaneous with what). Their tenseless theorist is also subject to our objection.

19 I take this example from Fine (2002: 165).

20 In Sect. 3.1 we gave a three-step argument that the qualitativist must take the global individualistic state to be grounded in the totality of qualitative facts taken together. We may now give a similar argument for the claim that the tenseless theorist must take the global tensed state to be grounded in the totality of tenseless facts taken together. First, the global tensed state is grounded in its conjuncts, the tensed facts, taken together. Second, the tenseless theorist is committed to taking the tensed facts *T* to be distributively grounded in the tenseless facts *L*. The argument of Sect. 3.1 appealed to the claim that any qualitative fact grounds some individualistic fact; here we may appeal to to the claim that any tenseless fact $L_{j}$ will ground some tensed fact. For instance, $L_{j}$ will ground the disjunction $L_{j}\vee S$, where *S* is an arbitrary tensed fact such as the fact that it is sunny. By chaining these two steps (as in Sect. 3.1) we obtain our conclusion.

21 If what one might call ‘qualitative translational symmetry’ counts as a genuine form of symmetry alongside other forms of translational symmetry, then one can mount the objection to the tenseless theory of time solely by appeal to the original symmetry principle, with no need for the variant. For in the world of eternal recurrence, the tenseless facts will possess qualitative temporal translational symmetry, while the tensed facts will not. I myself am not sure that this is indeed a genuine form of symmetry, and so I prefer the objection as developed in the text. Still, this alternative line of argument is worth considering. Thanks here to an anonymous referee.

22 He has since moved away from this version of comparativism. In his (2020), Dasgupta defends a version of comparativism which gives up the claim that the absolute facts are grounded in comparative facts and which instead regards absolutist statements like ‘the mass of my laptop is 1.25 kg’ as in a certain sense nonfactual. Whatever difficulties this nonfactualist version of comparativism may face, the objection developed in this section is not among them.

23 Dasgupta’s (2013) comparativism, like his qualitativism, is formulated in terms of a many-many relation of ground. On this formulation of comparativism, there is no need to appeal to the global absolute state.

24 Both of these forms of fragmentalism were developed by Fine (2005). Other discussions of tense-theoretic fragmentalism include Correia and Rosenkranz (2012), Lipman (2015), Hofweber and Lange (2017), and Loss (2017). Lipman (2016) develops a perspectival form of fragmentalism similar to Fine’s first-personal fragmentalism. I have followed Correia and Rosenkranz (2012) and Lipman (2015) in characterizing fragmentalism as the view that there are incompatible facts, while Fine (2005), on one reading, characterizes it as the view that there are incompatible facts all of which are fundamental. The contrast between these two characterizations is discussed at the end of this section.

25 From the fragmentalist’s point of view, Sect. 3.3’s grounding explanation of tensed facts in terms of tenseless facts involves only a proper subset of all of the tensed facts there are: those belonging to the $t_{0}$-fragment. Although the explanans of this explanation is symmetric, and its explanandum is asymmetric, there is no violation of the symmetry principle. For the explanation is not unpruned. After all, the tenseless facts explain not only the tensed facts of the $t_{0}$-fragment but those of all the other fragments as well. Once the explanandum is ‘depruned’ by including the other fragments, symmetry is restored.

26 It is briefly mentioned in Werner (2022).

27 My thanks to Erica Shumener, Ted Sider, and anonymous referees for their written comments on earlier drafts of this paper, as well as to Fatema Amijee, Fabrice Correia, Mahmoud Jalloh, Stephan Krämer, Roberto Loss, Michaela McSweeney, Asya Passinsky, Jan Plate, Mike Raven, Stefan Roski, Thomas Sattig, David Schroeren, Jon Simon, Jonas Werner, and audiences in Bristol, Buffalo, Hamburg, Ligerz, Norman, Tübingen, Vancouver, and at the Pacific APA. I am grateful for the support of the Program of Postdoctoral Fellowships at the Universidad Nacional Autónoma de México, the German Research Foundation (grant KR 4516/2-1), and the Swiss National Science Foundation (grant 197172).
